# New developments in Huntington’s disease and other triplet repeat diseases: DNA repair turns to the dark side

**DOI:** 10.1042/NS20200010

**Published:** 2020-11-16

**Authors:** Robert S. Lahue

**Affiliations:** Centre for Chromosome Biology and Galway Neuroscience Center, National University of Ireland, Galway, Newcastle Road, Galway H91W2TY, Ireland

**Keywords:** DNA synthesis and repair, genome integrity, mutation, neurodegeneration

## Abstract

Huntington’s disease (HD) is a fatal, inherited neurodegenerative disease that causes neuronal death, particularly in medium spiny neurons. HD leads to serious and progressive motor, cognitive and psychiatric symptoms. Its genetic basis is an expansion of the CAG triplet repeat in the *HTT* gene, leading to extra glutamines in the huntingtin protein. HD is one of nine genetic diseases in this polyglutamine (polyQ) category, that also includes a number of inherited spinocerebellar ataxias (SCAs). Traditionally it has been assumed that HD age of onset and disease progression were solely the outcome of age-dependent exposure of neurons to toxic effects of the inherited mutant huntingtin protein. However, recent genome-wide association studies (GWAS) have revealed significant effects of genetic variants outside of *HTT*. Surprisingly, these variants turn out to be mostly in genes encoding DNA repair factors, suggesting that at least some disease modulation occurs at the level of the *HTT* DNA itself. These DNA repair proteins are known from model systems to promote ongoing somatic CAG repeat expansions in tissues affected by HD. Thus, for triplet repeats, some DNA repair proteins seem to abandon their normal genoprotective roles and, instead, drive expansions and accelerate disease. One attractive hypothesis—still to be proven rigorously—is that somatic *HTT* expansions augment the disease burden of the inherited allele. If so, therapeutic approaches that lower levels of huntingtin protein may need blending with additional therapies that reduce levels of somatic CAG repeat expansions to achieve maximal effect.

The main purpose of this review is to provide an overview of recent developments in the role of DNA repair in Huntington’s disease (HD) and other triplet repeat diseases. The review is primarily intended for neurobiologists, physicians and others working in the field, or those interested in the general topic, but who are unfamiliar with DNA repair. For additional analysis, the reader is directed to other recent review articles [1–8].

## HD

HD (OMIM #143100) is named after George Huntington, an American physician who first reported its familial nature in 1872 [9]. HD typically affects adults [8], although there are cases of juvenile onset at ages as young as 2 years [10]. Initially, the major recognized symptom of HD was progressive chorea, usually presenting as involuntary and irregular movement of hips, shoulders and face [1]. Additional motor symptoms include failure of voluntary motor control (especially gait and swallowing), dystonia, bradykinesia and rigidity. However, it is now clear that the disease manifestations of HD are more complex. There are also substantial cognitive and psychiatric symptoms which often precede motor dysfunction, sometimes by many years [11–13]. Cognitive issues affect attention, memory and speech, which progress gradually to dementia [14]. Cognitive decline is a critical concern for HD patients and families, profoundly affecting quality of life [15]. Psychiatric symptoms are most often depression, anxiety, apathy and irritability, although some patients also exhibit aggression [11]. Together, the cognitive, psychiatric and motor symptoms lead to debilitating disease that typically lasts approximately 15 years and results in premature death. The gradual appearance of symptoms creates a challenge in unambiguously discerning age of onset, even for motor dysfunction, which is usually the most obvious change and is used to assign age of onset. As described later, the challenge in accurately identifying age of onset partially obscured the effects of genetic modifiers of disease.

Multiple brain regions are affected in HD [13,16], mainly due to neuronal death by apoptosis [17,18]. The striatum is the region affected most strongly, exhibiting gross atrophy. Medium spiny neurons are especially sensitive in HD [13], although the precise reason for this sensitivity remains obscure [19]. Disease progression is scored with the Vonsattel grading system of 0–4 based on striatal morphology of post-mortem brain [20]. The cerebral cortex is also visibly affected by neuronal loss and shrinkage [21]. Later disease stages also affect numerous other brain regions; because of these widespread effects, the brains of advanced stage HD patient weigh 25–30% less than normal [22]. Thus, advanced HD can be regarded as whole brain disease [13,16].

## HD genetics

Despite the complex nature of its symptoms, HD results from mutation of a single gene, *HTT* [23]. The mutation is autosomal dominant, meaning that one mutant copy of *HTT* leads to disease regardless of the presence of a second, normal allele. The worldwide HD prevalence is approximately 3 per 100000, but this value increases to ∼10 per 100000 in European and North American populations [24]. A well-known hotspot for HD is Lake Maracaibo, Venezuela with prevalence of 700 per 100000 [25], due to founder effects. Despite the devastation caused by HD, this Venezuelan population turned out to be a significant resource in mapping the mutated gene.

The genetic locus causing HD was mapped in 1983 to the tip of chromosome 4 [26]. The gene itself, now called *HTT*, was identified and sequenced in 1993 [23]. Its protein product is called huntingtin. Examination of *HTT* DNA sequence revealed a long CAG repeat in exon 1 whose expansion is now known to cause the disease. HD became the fourth triplet repeat expansion disease identified, after X-linked spinal and bulbar muscular atrophy (SBMA) [27], Fragile X syndrome [28–30] and myotonic dystrophy type 1 [31]. Normal individuals have 9–26 copies of CAG in *HTT*, with alleles of 17–20 repeats being most common [32]. Thus, the repeat itself is a natural part of *HTT* and only becomes problematic upon expansion. Individuals with 27–34 CAG repeats are not generally affected by HD but they are prone to passing longer repeat tracts to their offspring [33,34], a process known as genetic anticipation. A CAG range of 36–39 is pathogenic but exhibits reduced penetrance [35,36], meaning that not every individual in this range is affected. Alleles of 40 repeats or more are pathogenic with full penetrance [37]. Most adult-onset HD patients have alleles from 40 to ∼55 repeats. Alleles longer than ∼60 repeats are usually associated with the juvenile onset form of the disease [38]. For *HTT*, the CAG repeat falls in the coding region, where it encodes a polyglutamine (polyQ) tract [23]. HD is member of polyQ family of nine inherited diseases, which includes SBMA and several of the inherited spinocerebellar ataxias (SCAs) [6]. PolyQ diseases are protein-mediated, expressing a toxic protein that leads to disease [6].

One of the most remarkable features of HD is that its complex symptomology is due to addition of a few extra CAG repeats to one copy of one gene. An individual with 30 repeats is normal but another person with 40 repeats suffers the full scope of debilitating symptoms [37]. Surprisingly, this implies that adding a few more glutamine residues to those already present in huntingtin is the sole event necessary to cause this multisymptom disease. As described later, recent findings indicate that additional genetic changes in *HTT* are thought to add substantially to disease burden [39,40].

What aberrant properties does the mutant huntingtin protein convey? Among the many cellular and molecular changes that occur in the presence of mutant huntingtin [41], the protein causes transcriptional dysfunction, leading to altered expression signatures in many genes [41–43]. This disruption of normal expression patterns is one feasible explanation for the wide-ranging effects associated with the mutation. Both wildtype and mutant huntingtin are ubiquitously expressed in all tissues, including all brain regions albeit at different levels [44,45]. One function of the wildtype protein is in cellular trafficking [41]. It also contains domains called HEAT repeats that facilitate interactions with other proteins [41,46]. Proteomic analysis showed that mutant huntingtin is known to interact with hundreds of proteins [41,47]. One possibility is that the expanded polyQ tract exacerbates inappropriate protein–protein interactions, including the sequestration of transcription factors, which could explain part of the widespread transcriptional dysfunction. Mutant huntingtin is well known to cause persistent nuclear inclusions, which are aggregates visible by microscopy and containing many different proteins [48]. However, it is not clear that these inclusions are causal for HD or merely a byproduct.

One of the key demonstrations that the expanded *HTT* gene causing HD was the development of mouse models that closely mimic the human disease [49–52]. Initial experiments described a transgenic mouse with a fragment of the human *HTT* gene harboring exon 1 and the expanded CAG repeat. These animals developed progressive neurological phenotypes similar to the human condition [53]. The CAG repeat in these animals were also shown to be genetically unstable in both somatic tissues and during transmission to offspring [54]. Since these pioneering studies, a number of mouse models have been developed, including knock in animals, where the endogenous mouse *Htt* gene has been modified to harbor expanded CAG tracts [50]. Nearly all these mouse lines exhibit some or most disease aspects of HD, and most of them also show expansions of the CAG tract.

## Somatic expansions in *HTT* as a potential modulator of disease onset and progression

**Figure 1:**
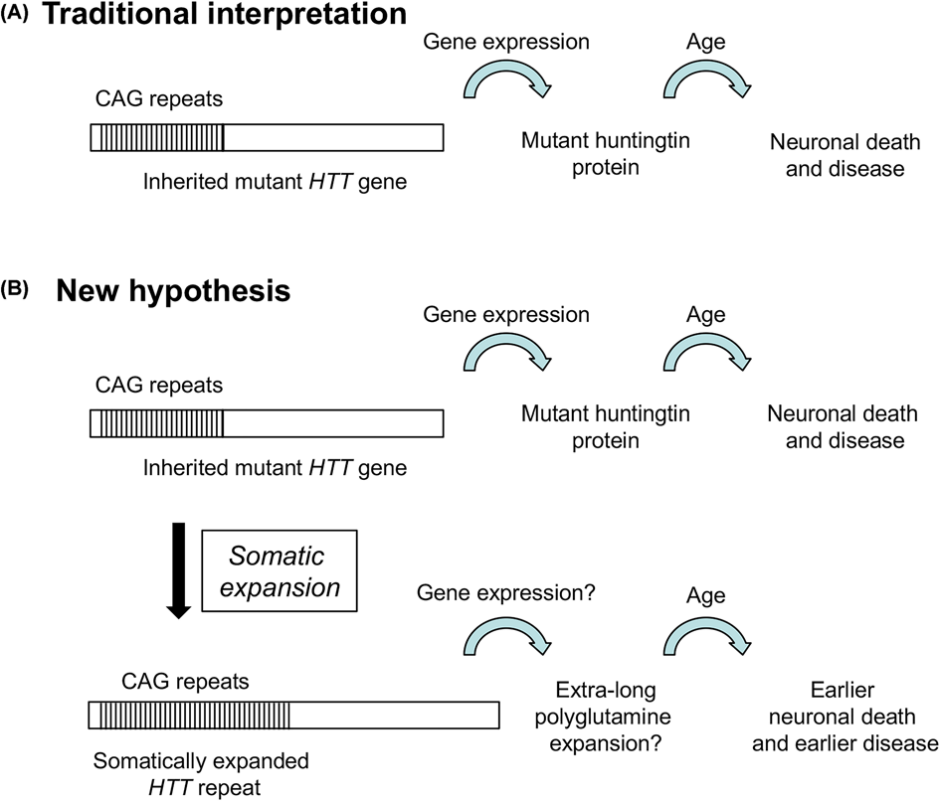
Models for neuronal death and HD (**A**) Traditional viewpoint of HD: inherited allele expresses mutant huntingtin protein. With age, neurons die due to toxic effect of mutant huntingtin. (**B**) New model. In addition to (A), ongoing somatic expansions add to toxic burden in neurons and other brain cells.

The traditional interpretation of HD is that exposure of neurons and other brain cells to the toxic mutant huntingtin over the course of years leads to age-related neuronal death (Figure 1A). This viewpoint is consistent with several key observations about HD. First, the CAG repeat expansion in *HTT* is the only mutation necessary to cause disease [23]. Second, the length of the inherited expansion is the major factor (∼60–70%) that determines age of disease onset [55–58]. Finally, mutant huntingtin exhibits toxic effects in many experimental systems [50,53]. However, the traditional viewpoint is less satisfactory in explaining why huntingtin with 30 glutamines is safe but protein with 40 glutamines is deadly. Also, there is significant evidence that ∼30–40% of the age of onset is determined by other features besides inherited CAG length [56–58]. What other elements should be considered?

One additional feature of HD is the existence of somatic expansions. In addition to inherited CAG repeat expansions, HD individuals also undergo somatic expansions that are now believed to impact disease onset and progression. In some somatic tissues, particularly in brain, the inherited *HTT* mutation continues to expand during the lifetime of the individual. Dramatic striatal expansions, up to 1000 repeats, were observed in HD brains [59,60]. Moreover, expansions were observed in striatal neurons of both humans and mice [61,62], where they occur early in the disease process and continue to accumulate [61].

What causes somatic instability and is it important? The inherited CAG length was shown to be a major modifier of somatic instability, although additional genetic and environmental modifiers were also deduced but not initially specified [55–58]. Somatic instability in *HTT* was shown to be a significant predictor of disease age of onset, with longer somatic expansions linked to earlier onset [63]. Together, these studies verified two key predictions of the hypothesis that somatic expansions modify HD onset and progression. First, somatic expansions occur in the tissues affected by HD, thus fulfilling the spatial requirement. Second, somatic expansions precede disease symptoms [60], supporting the temporal requirement. A third prediction is that genetic modifiers of somatic instability would also modify disease. These early groundbreaking studies helped lay the foundation for the recent identification of genetic modifiers of HD, described in the next section.

If somatic instability helps determine HD age of onset, then a new hypothesis for the disease can be formulated (Figure 1B) [2,5,8]. This new hypothesis retains the traditional thinking about the age-dependent toxic effects of inherited polyQ length in huntingtin but adds a second branch where somatic CAG repeat expansions hasten disease onset and progression. The somatically-expanded version of *HTT* is predicted to encode a mutant huntingtin protein with extra-long polyQ tract. By this model, the extra-long huntingtin adds to the disease burden and exacerbates onset and progression. This hypothesis can also be extended to the subset of SCAs where somatic expansions occur [2,7]. Somatic expansions are the linchpin of this model and recent genome-wide association studies (GWAS) pointed directly at DNA repair factors as novel modifiers of age of onset and progression of HD and in some of the SCAs.

## GWAS identify DNA repair factors as modulators of HD

GWAS search among a population for genetic variants that are associated with a particular trait, such as inherited disease. In the case of HD, GWAS sought factors that, by themselves, do not confer risk of HD but which modify the course of the disorder [3,39,40]. This approach is based on the idea that the expanded CAG repeat in HD patients provides a genetically sensitized background to find modifying factors [39]. The two major advantages of GWAS are first, that this approach makes no assumptions about disease mechanism. Therefore, GWAS is not influenced by any pre-existing expectation for factors that drive the outcome, in this case, HD age of onset. Second, GWAS takes advantage of existing information among HD cohorts of thousands of patients. In effect, nature has already performed the experiment and GWAS looks for the result. For HD, effective GWAS required the creation of large consortia of HD patients with accurate clinical and genetic information. TRACK-HD and Enroll-HD are examples of such consortia. Subsequent GWAS reports have also included information from several SCA consortia (SCA1, SCA2, SCA3, SCA6, SCA7 and SCA17) to extend the findings beyond HD [2,64]. Most HD GWAS have focused on age of motor onset (AOO) because it is quantifiable [39,40]. Additional GWAS reports stem from assessing disease progression [65] and CAG repeat instability in blood samples from patients [66].

The unexpected outcome from these GWAS is that DNA repair genes comprise many, although not all, modifiers for HD and other polyQ diseases [2,4,39,40,64–66]. Genetic loci were clearly identified that contain DNA repair genes *MSH3, MLH1, PMS1, PMS2, MLH3* and *FAN1*, which are described in more detail in the following section*.* The effect of polymorphisms in these loci was surprisingly large, accounting for alterations of up to 6 years in age of onset [39]. In addition, SNPs in some DNA repair genes are also associated with changes in progression of HD [67]. While the loci from GWAS often contain numerous genes and therefore cannot unambiguously identify a specific gene, a process called pathway analysis looks for commonalities among candidate genes in independent loci. The pathway analysis is clear that DNA repair, and particularly one particular activity called DNA mismatch repair (MMR), is very tightly associated with AOO, providing strong secondary proof of the correct gene assignments [39]. A third correlation is that polymorphisms that favor MMR expression accelerate HD age of onset, whereas alternative SNPs that reduce expression slow AOO [65,67]. Thus, GWAS supports the idea that high levels of MMR proteins and repair activity accelerate disease onset in HD. A fourth point is that HD onset tracks with CAG repeat length, not the number of glutamine codons [40,68]. This finding helped refocus attention from huntingtin back to the *HTT* DNA itself. A final supporting line of evidence is that DNA repair factors in HD mice are well known to influence somatic CAG repeat expansions [69] and to modulate disease [70]. Knockouts of *Msh2* or *Msh3* (encoding MutSβ) [70–74] or of *Mlh1* or *Mlh3* (encoding MutLα and MutLγ) [75] eliminate nearly all somatic and inherited *Htt* expansions. In total, this evidence provides a very strong case that at least some modulation of HD occurs at the level of maintaining the *HTT* DNA itself [4].

## DNA repair loci implicated in HD and other triplet repeat expansion disorders

The strong connection between DNA repair and HD age of onset and disease progression has led to significant new thinking about maintenance of the CAG repeat within *HTT* as a major modifier of disease. This DNA-centric view has major implications for both scientific mechanism, which is considered in this section, and therapeutic approaches to HD and related polyQ diseases that are considered later.

**Figure 2:**
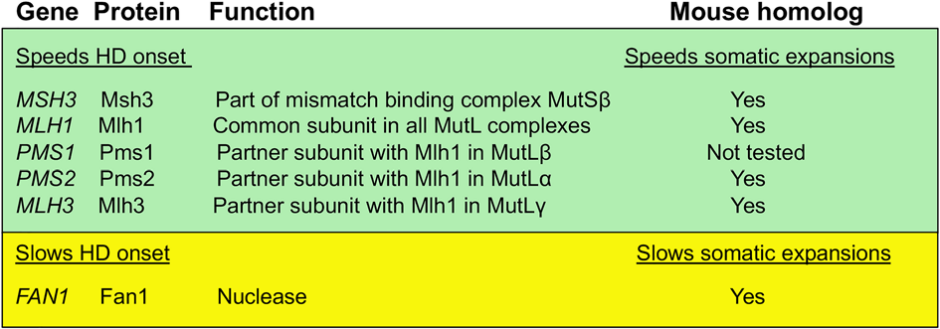
DNA repair genes and their proteins identified by GWAS

The relevant DNA repair genes and the functions of their proteins are summarized in Figure 2. The first panel includes factors that speed onset of HD. *MSH3* encodes a protein that identifies DNA damage, specifically mismatched DNA that normally arises from errors in DNA replication. The Msh3 protein partners with a related but distinct protein called Msh2 to form the functional complex, MutSβ [76]. Both Msh3 and Msh2 are required for MutSβ activity. Additional MMR proteins are encoded by *MLH1, PMS1, PMS2* and *MLH3* (Figure 2), referred to collectively as the MutL homologs, after the bacterial prototype. The eukaryotic MutL proteins function as heterodimers, with Mlh1 protein being the common partner. Inclusion of either Pms2, Pms1 or Mlh3 yields MutLα, MutLβ and MutLγ, respectively [76]. Mouse studies identified a key role in expansions for both MutLα [77] and MutLγ [75]. While each of these MutL complexes has unique roles in MMR, a recent finding in cultured mouse Fragile X stem cells surprisingly suggested that all three MutL complexes are required for CCG repeat expansions [78]. It is not yet known if all three MutL complexes are also required for expansions of CAG repeats. The potential mechanistic role of MMR in expansions is considered in the next section.

GWAS analysis also identified a prominent signal for another gene, *FAN1*, whose protein product Fan 1 acts independently of MMR (Figure 2). GWAS analysis suggest that the key SNP in *FAN1* is associated with high expression of the Fan1 protein and is also associated with later age of onset. Thus, in contrast with MMR which accelerates disease, the presence of Fan1 acts in a disease-slowing process [39,40,79]. Fan1 is a nuclease, an enzyme that cleaves DNA in a distinct pathway called interstrand cross-link repair. Mouse and stem cell knockout studies of *FAN1* are consistent with a protective role for Fan1 in blocking triplet repeat expansions [80–82]. One possibility is that Fan1 might remove DNA intermediates before they become fully expanded, thereby stabilizing the repeat sequence. A recent mouse study looking at double knockouts of *Fan1* and *Mlh1* found that functional Mlh1 protein was required to see the CAG repeat destabilization that occurs due to *Fan1* knockout [83], suggesting that normally Mlh1 is required to allow Fan1 stabilization of the triplet repeat. This effect could be due to protein–protein interactions that were reported between Fan1 and the MutL homologs Mlh1, Pms1, Pms2 and Mlh3 [84].

**Figure 3:**
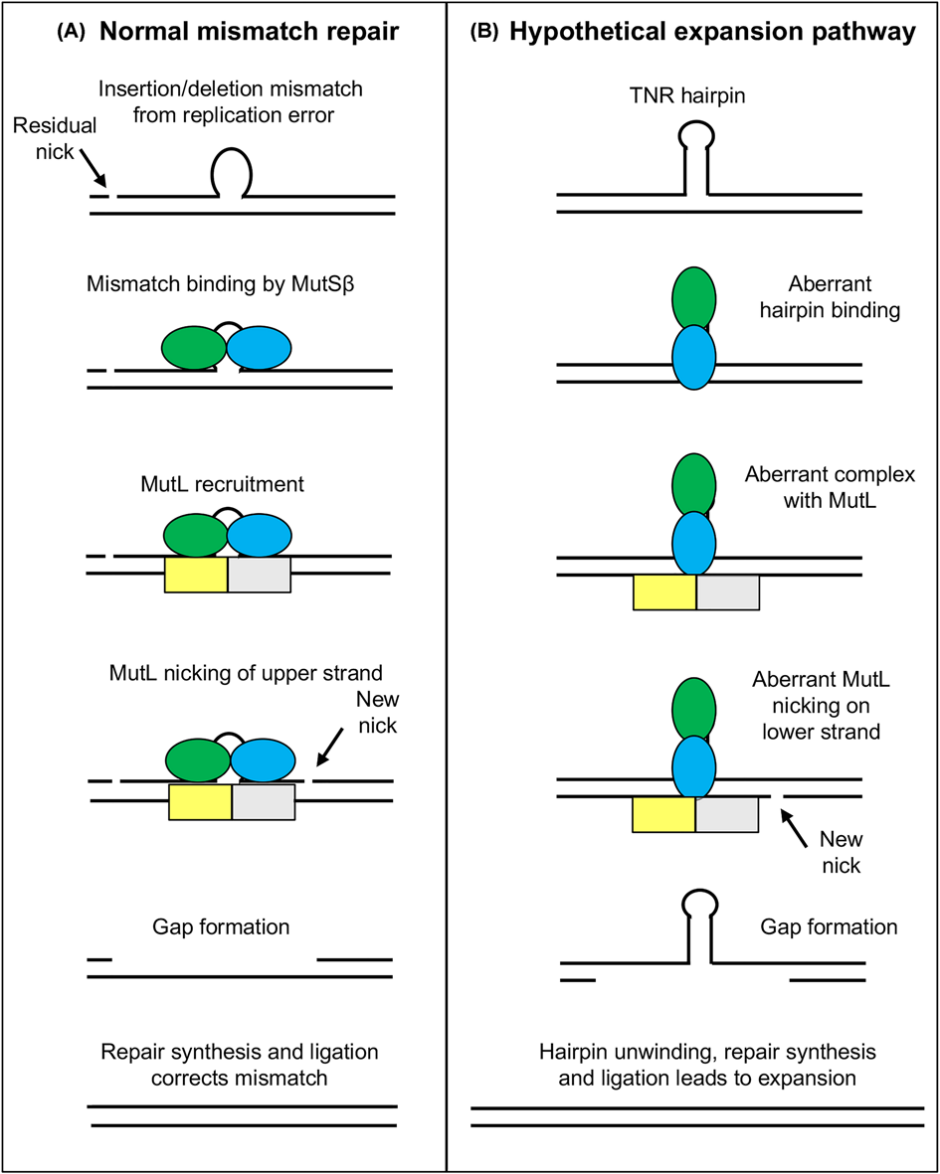
Model for how non-canonical DNA MMR could drive expansions (**A**) A simplified scheme for how normal MMR correctly repairs ‘top’ strand to remove replication error. *Ovals*, MutSβ subunits Msh2 (green) and Msh3 (blue). *Rectangles*, MutL homolog subunits Mlh1 (yellow) and either Pms2 or Mlh3 (grey). (**B**) Hypothetical scheme for how non-canonical MMR aberrantly repairs ‘bottom’ strand to cause expansion.

What does MMR normally do and what goes wrong to cause triplet repeat expansions? The major role of MMR is as a genetic spellchecker to correct errors made during DNA replication (Figure 3A) [76,85–87]. Although replication is very precise, the size of mammalian genome leads to inevitable errors. For human cells, a few hundred mismatches or so are left behind after each round of replication [88]. Some of these mismatches are in repetitive sequences and involve synthesis of too many or too few copies of the repeat, referred to as insertion/deletion mispairs (loop symbol in Figure 3A). The MMR protein MutSβ recognizes these insertion/deletion mismatches and triggers their repair [89]. Thus, MutSβ provides the first key function in MMR, finding the ‘needle’ (mismatch) per ‘haystack’ of ∼1 million correctly synthesized base pairs. The second required step in MMR is identifying which strand has the incorrect information and therefore must be targeted for repair. This is primarily the function of the MutL homolog proteins [90]. Since both strands of the mismatch comprise normal Watson and Crick bases—A, C, T and G—there is no chemical signal to direct MMR to the strand with the incorrect sequence. Instead, MMR uses residual strand breaks (nicks) left over from DNA replication to identify the newly synthesized strands (Figure 3A) [91,92]. Using protein–protein interactions between the MutS and MutL homologs and other factors, the nicks are used to direct repair to the strand bearing the incorrect information [93]. In some repair events, the MutL proteins introduce a second nick into the newly synthesized strand (Figure 3A) [94]. Subsequent processing by additional nucleases creates a single-strand gap that removes the mismatch and a few hundred neighboring base pairs. The gap is then filled by DNA synthesis and the strand is sealed by DNA ligase to complete the repair process [94].

There are several key differences in MMR activity on triplet repeats (Figure 3B). First, mismatched TNRs are known to form DNA secondary structures, such as hairpins (stem-loops) [95]. Hairpins can occur in post-mitotic neurons, since hairpin formation does not require DNA replication. Instead, experiments in model systems show that hairpins can occur during gene transcription. The requirement for strand separation during transcription allows transiently single-stranded TNR DNA to fold on itself prior to reannealing with the complementary strand [96–99]. Biochemical experiments with model DNA substrates showed that MutSβ binds significantly differently to TNR hairpins compared with normal insertion/deletion mispairs [73,100]. Although not to be taken literally, this difference in binding is shown schematically as a ‘vertical’ alignment of MutSβ proteins (Figure 3B). Second, somatic expansions occur in postmitotic neurons [61,62] where there is no DNA replication. This means that there is no nick to serve as a strand signal for MMR. Instead, this model suggests that MutL homolog proteins interact aberrantly with the unusual TNR hairpin–MutSβ complex. This aberrant interaction leads to MutL-mediated incision of the DNA on the incorrect strand, across from the hairpin [101,102] (Figure 3B). Subsequent processing of the nick by additional nucleases opens up a gap, which is then filled in by DNA repair synthesis and sealed by a DNA ligase. The overall outcome, in this hypothetical situation, is to cause an expansion (Figure 3B). The model is also consistent with the fact that DNA repair is functional in non-dividing cells [4]. Finally, this model has the virtue of simplicity—it only requires that MMR incises the wrong strand, but otherwise utilizes most of its normal mechanism. An interesting challenge for DNA repair experts will be to develop assays to test this model.

## Post-GWAS: a new model for understanding HD age of onset and progression

The important revelations about somatic CAG repeat expansions in *HTT* suggest that the traditional model of disease may need refinement to add somatic expansions as part of the pathogenic process (Figure 1B). While neurons with the inherited mutant *HTT* gene continue to express mutant huntingtin with its toxic effects, any cells with a somatic expansion are predicted to express an extra-long version of mutant huntingtin protein with even more glutamines than encoded by the inherited allele. If huntingtin with extra glutamine residues is more toxic than the inherited version, then somatic expansions would add to neuron toxicity and therefore cause earlier neuronal death and disease pathogenesis. This is one model to explain the GWAS findings (Figure 1B). A competing model is that neurons with inherited expansions undergo exposure to DNA damaging agents, such as oxidative damage, or to toxicity induced by somatic expansions. By this model, the key role of DNA repair protects neurons after damage. Genetic variants in DNA repair are predicted to result in sensitization to this damage, leading to earlier cell death [5]. The key difference is that model 1 predicts that somatic expansions contribute significantly to disease, whereas model 2 does not. The best available data to distinguish the two models used an HD mouse model in which a DNA repair gene called *OGG1* was inactivated [103]. Although *OGG1* has not shown up in GWAS reports on triplet repeat diseases, the gene is known to promote somatic CAG repeat expansions in some mouse models of HD [103,104] and therefore provides a useful experimental tool. Loss of *OGG1* in this system selectively block somatic expansions, and these animals showed a delay in onset of HD-like symptoms compared with control littermates. While these results support model 1, they can still be interpreted in light of model 2.

An even better test to distinguish model 1 from model 2 would be to create an HD mouse model with an inherited expansion that encodes mutant huntingtin but where the *Htt* gene cannot undergo somatic expansions. If model 1 is correct, disease onset should be slowed due to loss of the somatic expansions, whereas model 2 predicts no change in disease onset. How could such an experiment be designed? One way is to take advantage of the genetic redundancy in glutamine codons. Both CAG and CAA encode glutamine, but normally the human *HTT* sequence is nearly all CAG codons, with a CAA codon or two near the 3′ end [66,68,105]. Artificially changing this sequence to include more CAA codons (‘interruptions’) scattered through the repeat would still encode huntingtin with the same number of glutamines. However, interrupted triplet repeats are known to be much more genetically stable, with fewer expansions [40,66,68]. The prediction of this experiment is that the interrupted version of mouse *Htt* would retain the ability to encode the inherited form of huntingtin, but the gene itself would undergo few somatic expansions and thereby greatly reduce abundance of any putative ‘extra-long’ polyQ huntingtin. If model 1 is correct, disease onset should occur later in mice with the interrupted *Htt* gene compared with animals with the uninterrupted version. Model 2 predicts no significant difference in disease onset. The idea was tested in one mouse model where the polyQ tract of huntingtin is encoded by mixed CAA-CAG repeats and is genetically stable. The result suggests that, in this mouse model, somatic instability does not play a necessary role in the selective neuropathogenesis [106]. Unfortunately, no comparison was available with a perfect CAG repeat control animal, suggesting that this result should be viewed with caution.

HD homozygotes provide another interesting evaluation of model 1. Although rare, some individuals harbor two mutant *HTT* alleles, which can be of different CAG repeat length. Age of disease onset correlates with the longer of the two alleles [68,107]. If model 1 is correct, why does the shorter expanded allele seem not to have much effect on age of onset? One possibility is that the longer allele is primarily targeted for somatic expansions, perhaps due to the greater number of repeats. A useful experiment would be to look for allele-specific somatic expansions and see if they primarily stem from the longer allele. A second possibility is that both alleles undergo somatic expansions at similar frequencies but that disease onset is somehow particularly sensitive to changes in the longer repeat tract. Testing these possibilities and any other theories will require additional experimentation.

## Connections to therapy

**Figure 4:**
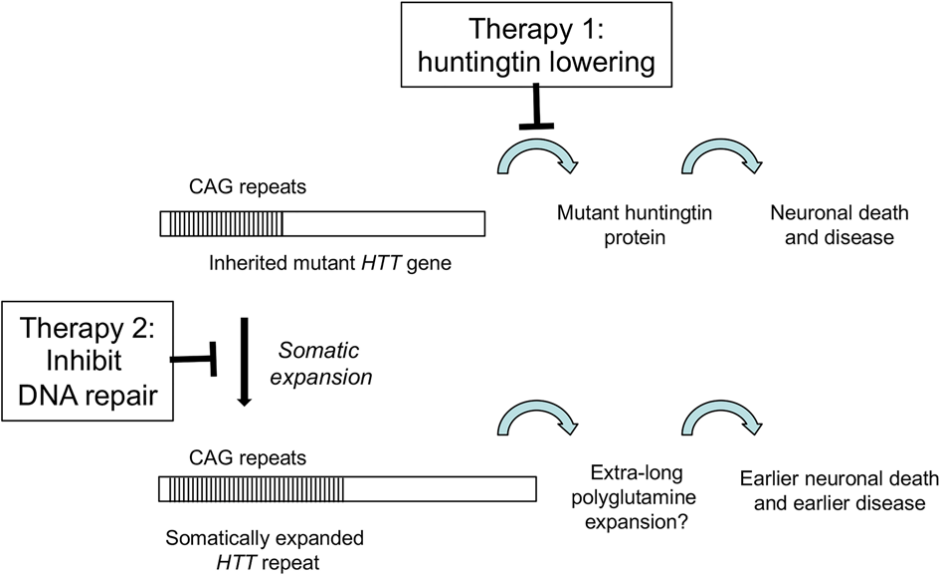
Potential therapies against HD Therapy 1 involves huntingtin lowering strategies. Therapy 2 seeks to inhibit specific DNA repair factors to reduce somatic CAG repeat expansions.

How might this new information about DNA repair affect therapeutic efforts for HD and other triplet repeat expansion diseases? A major effort is currently underway to treat HD by lowering the levels of huntingtin protein [108,109] (Figure 4). The idea is that less huntingtin—especially less of the mutant version of the protein—will reduce HD symptoms and relieve suffering. As an example, antisense oligonucleotides (ASOs) have been developed that inhibit translation of huntingtin by targeting its messenger RNA [108,109]. One such ASO was reported in a Phase I/IIa clinical study to be safely tolerated and to reduce huntingtin levels in spinal fluid by up to 40% [110]. This ASO is now proceeding to a Phase III trial. These huntingtin-lowering approaches are a welcome addition and hopefully they will prove safe and efficacious for HD patients. In principal, the protein-lowering approach could be used to reduce other expanded polyQ proteins that cause additional diseases [111]. An obvious drawback is that individual ASOs must be designed and tested for each disease. A second unanswered question is whether ASO technology would be effective against the putative ‘extra-long’ version of *HTT*. While there is no reason *a priori* to believe otherwise, this point will require experimental proof.

A second approach to therapy was opened by the discovery that genetic variants in DNA repair proteins, particularly MMR proteins, help drive disease [39,40]. The possibility that somatic expansions are important for disease onset and progression means that MMR proteins might be additional druggable targets that would impact HD and any other triplet repeat expansion diseases with somatic instability (Figure 4) [112]. As an example, supportive evidence from an HD mouse model indicates that knockout of the *MSH2* subunit of MutSβ eliminated striatal expansions and also delayed nuclear accumulation of mutant huntingtin [70,113]. Since the mice contain ∼111 uninterrupted CAG repeats, this result suggests effectiveness against the putative extra-long version of mutant huntingtin alluded to above. Another reason to be attracted to this idea is that loss of *MSH3*, which encodes the unique subunit of MutSβ, has a low impact on cancer predisposition [106]. Thus, inhibiting MutSβ to treat triplet repeat expansion diseases may minimize any complications in tumorigenesis. This line of thinking has led to vigorous efforts to identify and test MutSβ inhibitors for efficacy in HD. Several entities have initiated very active efforts to screen for small molecules that inhibit MutSβ (for example, see https://chdifoundation.org/dna-repair-handling/). In principle, these interventions could target MutSβ in several ways [108]. One way is to inhibit its enzymatic activity, particularly the ATPase function that is crucial for expansions [114], perhaps using novel small molecule inhibitors that would necessarily need to be selective for MutSβ. A second approach would be to disrupt the interactions of MutSβ with the MutL homologs. This will require clear identification of which MutL homolog is most important for driving expansions, development of a clear understanding of the relevant protein–protein interactions and a suitable screen for disrupting agents with good specificity. A third idea is to reduce the abundance of Msh3, one of the subunits of MutSβ, through ASO or similar technology. Perhaps ASO against Msh3 could be added to the huntingtin ASOs already being tested. Studies in mice and human cells show that lower Msh3 abundance leads to fewer CAG repeat expansions [114–117]. Msh3 levels in humans also correlate positively with disease progression [65,67]. A final approach, which already has some positive preclinical support, is to inhibit enzymes that activate MutSβ. One such enzyme is the histone deacetylase, HDAC3, which was recently demonstrated to directly deacetylate MutSβ and stimulate expansions [118]. Potent inhibitors already exist that selectively block HDAC3 activity [119–123]. Several of these HDAC3 inhibitors have been shown in mouse studies to alleviate motor and cognitive symptoms of HD, and also to inhibit striatal *Htt* expansions [120,124,125]. Any of these approaches can, in principal, be blended with huntingtin-lowering therapy to potentially provide a more potent therapy against HD and related triplet repeat expansion diseases.

## Conclusions and future perspectives

It was a revelation when GWAS identified DNA repair proteins as modifiers of HD age of onset and disease progression [2,4,39,40,65,66]. Perhaps the biggest surprise was the extent of this modification—polymorphisms in DNA repair genes can mean the difference of up to 6 years of healthy living for HD patients [39]. Moreover, the modifier genes discovered by GWAS were highly consistent with mouse studies and cellular experiments that had identified many of the same DNA repair proteins as causal for somatic CAG repeat expansions. Together, these findings open two important avenues for ongoing studies. The first is mechanistic: do somatic expansions add to disease burden in HD and, if so, how? The second is therapeutic: regardless of the mechanism of how DNA repair modifies HD, can DNA repair be used as a therapeutic target? Both these avenues offer exciting new opportunities to better understand HD and some of the related triplet repeat diseases, and they open a potential new therapeutic landscape for what had been untreatable conditions.

## Funding

The author is a paid consultant of LoQus23 Therapeutics and serves on their scientific advisory board; and Science Foundation Ireland [grant number 16/BBSRC/3395].

## Abbreviations

AOO: age of motor onset
ASO: antisense oligonucleotide
GWAS: genome-wide association studies
HD: Huntington’s disease
HEAT: huntingtin/elongation factor 3/protein phosphatase 2A/Tor1
MMR: mismatch repair
polyQ: polyglutamine
SBMA: X-linked spinal and bulbar muscular atrophy
SCA: spinocerebellar ataxia
TNR: trinucleotide repeat
